# Low Protein Diet Reduces Proteinuria and Decline in Glomerular Filtration Rate in Advanced, Heavy Proteinuric Diabetic Kidney Disease

**DOI:** 10.3390/nu16111687

**Published:** 2024-05-29

**Authors:** Liliana Garneata, Carmen-Antonia Mocanu, Tudor Petrisor Simionescu, Andreea Elena Mocanu, Diana Ramona Dragomir, Gabriel Mircescu

**Affiliations:** 1 Department of Internal Medicine and Nephrology, “Carol Davila” University of Medicine and Pharmacy, 050474 Bucharest, Romania; 2 Department of Nephrology, “Dr. Carol Davila” Teaching Hospital of Nephrology, 010731 Bucharest, Romania

**Keywords:** chronic kidney disease, low-protein diets, diabetic kidney disease, management of CKD, dietary intervention in CKD

## Abstract

Low protein diet (LPD) seems beneficial in ameliorating the complications of chronic kidney disease (CKD), in reducing proteinuria and the decline in kidney function, thus postponing the need for kidney replacement therapy (KRT). However, this type of intervention was less investigated in diabetic kidney disease (DKD). This is a single-center, prospective, interventional study that aims to assess the efficacy of reducing proteinuria and the rate of decline in the estimated glomerular filtration rate (eGFR). Patients with advanced DKD (stable proteinuria > 3 g/g and eGFR < 30 mL/min) with a good nutritional status and accepting a LPD were evaluated for inclusion. Ninety-two of the 452 screened patients (66% males, median age 61 years, proteinuria 4.8 g/g creatininuria, eGFR 11.7 mL/min/1.73 m^2^) completed the study. Intervention consisted of LPD supplemented with ketoanalogues of essential amino acids (KA) along with conventional nephroprotective therapy. Efficacy parameters were the variation in proteinuria and in eGFR from baseline to the end of the study. Proteinuria decreased 3-fold, and the rate of decline in eGFR decreased 5-fold in the intervention phase. No patient initiated KRT or died. LPD supplemented with KA seems effective in safely postponing KRT by reducing proteinuria and the decline in kidney function in advanced DKD.

## 1. Introduction

Diabetic kidney disease (DKD), defined as chronic kidney disease (CKD) in patients with diabetes mellitus (DM) [1,2], is the leading cause of kidney replacement therapy (KRT) initiation all over the world [3]. Moreover, DKD increases not only the kidney risk but also the cardio-vascular risk [4].

The updated “KDIGO practice guidelines for diabetes management in CKD” recommend a “comprehensive, holistic, approach to patient care” and place the nutritional intervention on the first “layer” of care. An individualized diet rich in vegetables, fruits, whole grains, and poor in processed meat (protein intake of 0.80 g/kg/day) and salt (<5 g/day) is recommended [5,6]. Moreover, the “KDOQI Clinical Practice Guidelines for Nutrition 2020” suggests a restriction in protein intake to 0.60–0.80 g/kg/day in patients with diabetes and CKD 3–5 [7].

In non-diabetic patients, low and very low protein diets (LPD, VLPD), supplemented with ketoanalogues of essential amino acids (KA) or not, postponed KRT initiation not only by improving the metabolic complications of advanced CKD, but also by reducing proteinuria and the decline in glomerular filtration rate (GFR) and by better controlling blood pressure [8,9]. Notably, very low-protein diets and adherence to the full KA dose had better results [10].

Implementation of an LPD in patients with diabetes mellitus is challenging because of the difficulties in achieving the energy intake (with presumed risk of malnutrition) and the low patients’ acceptance. Consequently, the clinical experience with an individualized approach to low-protein diets, either mostly vegetarian or mixed, is limited in DKD.

Some studies and a meta-analysis suggest an impressive reduction in the rate of eGFR decline of 5.82 mL/min but not in proteinuria in DKD patients on such diets [11,12,13]. However, these studies enrolled few diabetic patients with high-risk CKD: eGFR was higher than 60 mL/min, and only a few of them had a urinary protein/creatinine ratio higher than 500 mg/g. In addition, few studies examined the utility of LPD supplementation with KA in patients with high-risk DKD [14], although the effect of supplementation seemed beneficial [15]. However, protein-restricted diets proved to be nutritionally safe [16].

On the other hand, proteinuria—driven by hyperfiltration—is important, both pathogenetically and clinically, in DKD. As the intake of protein of animal origin increases hyperfiltration, it has been suggested, but not proven, that low-protein diets might be beneficial by reducing hyperfiltration.

Therefore, we intended to evaluate the efficacy, safety, and feasibility of a low-protein diet supplemented with ketoanalogues of essential amino acids in patients with type 2 diabetes mellitus and very high-risk CKD (proteinuria >3 g/g creatinine and eGFR < 30 mL/min/1.72 m^2^).

## 2. Materials and Methods

### 2.1. Design

**Figure 1:**
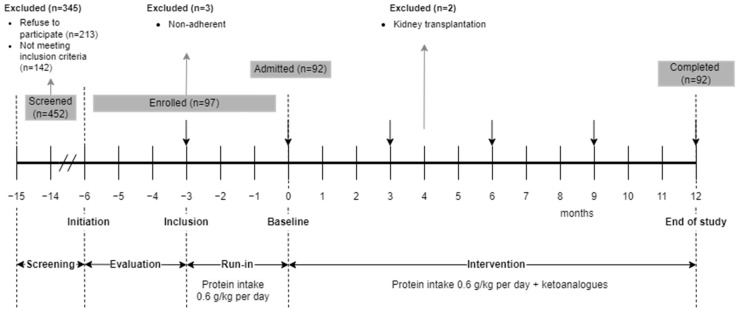
Study design.

This is an interventional prospective, unicentric, uncontrolled study with a 15-month total duration and four phases (Figure 1).

In the screening phase, all the patients with type 2 DM consecutively admitted for CKD in a tertiary department of nephrology were evaluated for enrollment. The patients who met the selection criteria and accepted the nutritional intervention signed the informed consent and entered a 3-month evaluation phase when variations in eGFR were assessed.

In the run-in phase, the enrolled patients received intensive nutritional counseling and were placed on a mostly vegetarian, low-protein diet. Adherence to LPD was monitored every 2 weeks in the first month, then monthly. Only patients with documented adherence to LPD were included.

In the intervention phase (12 months), the low protein diet was supplemented with KA. The patients were monitored monthly (Figure 1). Conventional kidney protective therapy and treatment of CKD complications continued under the in-charge physicians’ supervision as per current guidelines.

A sub-analysis of this study data referring to the relationships between salt intake, protein intake, blood pressure, and kidney and cardiovascular outcomes was previously published [17].

### 2.2. Selection Criteria

Adult patients (>18 years) with type 2 DM and very high-risk CKD [18]—category G4+ (eGFR < 30 mL/min/1.73 m^2^), MDRD4 equation [19] and category A3 (proteinuria > 500 mg/g)—were considered for inclusion.

The inclusion criteria were: urinary protein to creatinine ratio > 3 g/g and eGFR < 30 mL/min, both stable (variation less than ±5% during the evaluation phase), good nutritional status (Subjective Global Assessment Score—SGA A [20], serum albumin > 3.5 g/dL), and strict compliance to prescribed protein intake (estimated protein intake ±10% of prescribed) during the run-in phase.

Patients with other active kidney disease demanding specific therapy (recent increase in proteinuria to nephrotic range, acute decrease in eGFR, dysmorphic hematuria), absence of diabetic microangiopathy, those with severe comorbidities (heart failure, peripheral artery disease, liver cirrhosis, malabsorption, any active infection, inflammatory disorders requiring corticosteroids or immunosuppressive therapy, and those with uremia (pericarditis, gastrointestinal disorders, bleeding) were excluded.

Of the 452 screened patients, 213 refused to participate, and 142 did not meet the selection criteria. Ninety-seven patients (21% of 452) were enrolled in the run-in phase. Three out of them (3% of 97) did not correctly follow the LPD and were excluded. Two out of 97 (2%) pre-emptively received a kidney graft in the evaluation phase. All 92 remaining patients (20% of those screened) completed the study (Figure 1).

### 2.3. Intervention

The intervention consisted of a mainly vegetarian, low-protein diet (0.6 g/kg/day). To increase adherence, less than 5 meals of animal-derived food per week were allowed, no more than one per day. The choice of vegetables, fruits, legumes, and cereals was free. The diet was supplemented with KA (Ketosteril™ Fresenius Kabi, Bad Homburg, Germany), 1 tb/10 kg-dry weight-day, t.i.d., during meals.

The recommended daily energy intake was 30 kcal/kg-dry ideal body weight. Salt intake was also restricted to 5 g/day.

Patients received intensive nutritional counseling during the run-in phase and were instructed to keep a 3-day food diary. The nutritional counseling was further intensified with constant feedback to the 3-day food diary: once every other week in the first month, monthly for the next 3 months, and every three months thereafter.

### 2.4. Parameters and Measurements

The main efficacy parameters were proteinuria and eGFR variation from baseline to the end of study (EOS).

Proteinuria was measured in a 24 h urine collection and expressed as g/g creatinine. eGFR was estimated based on serum creatinine, age, sex, and ethnicity using MDRD4 variables equation [5]. Seum creatinine was measured enzymatically by the sane traceable method throughout the study.

Systolic and diastolic blood pressure were measured according to the ESH-EHC guidelines [21] and the mean arterial blood pressure (MAP) was computed as MAP = DBP + 1/3(SBP − DBP). Hypertension was defined as either BP over 140/90 mmHg or antihypertensive medication. Uncontrolled hypertension was defined as MAP over 97 mmHg (equivalent to the recommended target of 130/80 mmHg [7,21].

The safety parameters were related to the nutritional status—energy intake, SGA, body mass index (BMI), serum albumin, and C-reactive protein (CRP)—and to the glycemia control (glycated hemoglobin—HbA1c).

Patients’ adherence to the diet was evaluated by urinary urea excretion [22] and by an estimation based on the 3-day food diary for energy intake. Compliance was defined as a difference between the achieved and recommended levels of protein and/or energy of less than ±10%.

Daily carbohydrate intake and antidiabetic medicines were prescribed by the attending diabetologists.

The attending nephrologists were allowed to freely adjust antihypertensive medication—angiotensin converting enzyme inhibitors or angiotensin receptor blocking agents, calcium channel, and beta-blockers as well as loop diuretics (furosemide)—targeting a 130/80 mmHg BP and reducing proteinuria [7,21]. Renin–angiotensin–aldosterone system inhibitors (RAASi) and furosemide prescriptions were recorded at each visit. No patient received SGLT2 inhibitors.

The complications of advanced CKD were managed in accordance with current guidelines [5,18].

Uremic symptoms—pulmonary edema, acid-base or electrolytes disorders impossible to control by conservative management, malnutrition, anorexia—were criteria to initiate KRT. The decision to initiate KRT was made by the Hospital Ethics Committee, considering each patient’s clinical condition, independent of investigators.

Data obtained at initiation, inclusion, baseline, 3, 6 and 9 months, and at EOS were used for analyses.

### 2.5. Statistical Analysis

Continuous parametric variables are presented as mean or median with confidence intervals (95% CI), according to distribution. The distribution was evaluated with the Shapiro–Wilk test. Categorical variables are presented as percentages.

Comparisons were evaluated by t-Student, Wilcoxon, Mann–Whitney and Tukey–Kramer comparisons test for continuous variables and Pearson’s Chi^2^ or Z-score for the nominal ones, respectively.

Slopes of relevant parameters were calculated by linear regression, using values observed at study moments.

The factors associated with proteinuria were estimated in a model of linear logistic regression, using transformed variables to optimize the accuracy of analyses.

Differences were considered statistically significant at a *p* value of 0.05.

The statistical analysis software used for this study were Analyze-it ver.6 (Analyze-it Software, Ltd., Leeds, UK) and IBM SPSS ver. 25 (IBM, New York, NY, USA).

### 2.6. Ethics

The study was approved by the local Ethics Committee and registered in the National Clinical Trials database (NCT 03415074).

An analysis of data concerning the effects of the diet on blood pressure was already published [17].

## 3. Results

**Figure 2:**
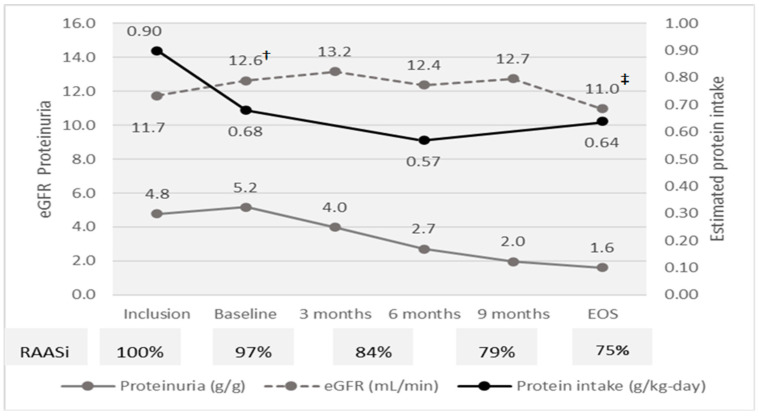
Efficacy parameters and compliance to diet at study moments. eGFR † Inclusion vs. Baseline *p* < 0.0001; ‡ Baseline vs. EOS *p* < 0.0001.

At inclusion, the median age was of 61 years and most patients were males (66%). The median eGFR was 11.7 mL/min and proteinuria was heavy (4.8 g/g). The median MAP was 98 mmHg under therapy with RAASi (85%) and furosemide (52%). Most patients were overweight (BMI 27.3 kg/m^2^) and all had SGA A. Median serum albumin was 3.9 g/dL, and inflammation was moderate (C-reactive protein 13 mg/L). The estimated protein intake was 0.89 g/kg-day (Figure 2; Table 1).

In the run-in phase, eGFR slightly increased, while proteinuria did not change. The proportion of patients with controlled BP did not change, but the proportion of patients treated with RAASi and furosemide increased with 15% and 10%. The protein intake decreased from 0.89 to 0.68 g/kg-day. BMI decreased with a median of 0.8 kg/m^2^, while serum albumin and CRP did not change, and the glycemia control improved (Hb1c decreased with 0.4% (Figure 2; Table 1 and Table 2).

### 3.1. Efficacy Parameters

#### 3.1.1. Estimated Glomerular Filtration Rate

The eGFR decreased during the evaluation phase by 1.5 mL/min (*p* = 0.01) and increased by 0.9 mL/min (*p* = 0.05) in the run-in phase, probably related to the reduction in creatinine pool due to lower animal meat related creatine intake. Thereafter, eGFR was steady in the intervention phase until month 9, when it decreased to EOS by −1.8 (−2.6 to −1.0) mL/min; *p* < 0.0001. In the intervention phase, eGFR decreased by −1.7 (−2.5 to −0.8) mL/min (*p* < 0.0001). Patients treated with RAASi at any moment of study had similar eGFR 12.8 (12.3 to 13.1) versus 11.5 (8.7 to 17.2); *p* = 0.24, suggesting that RAASi had little influence on eGFR variation (Figure 2).

**Figure 3:**
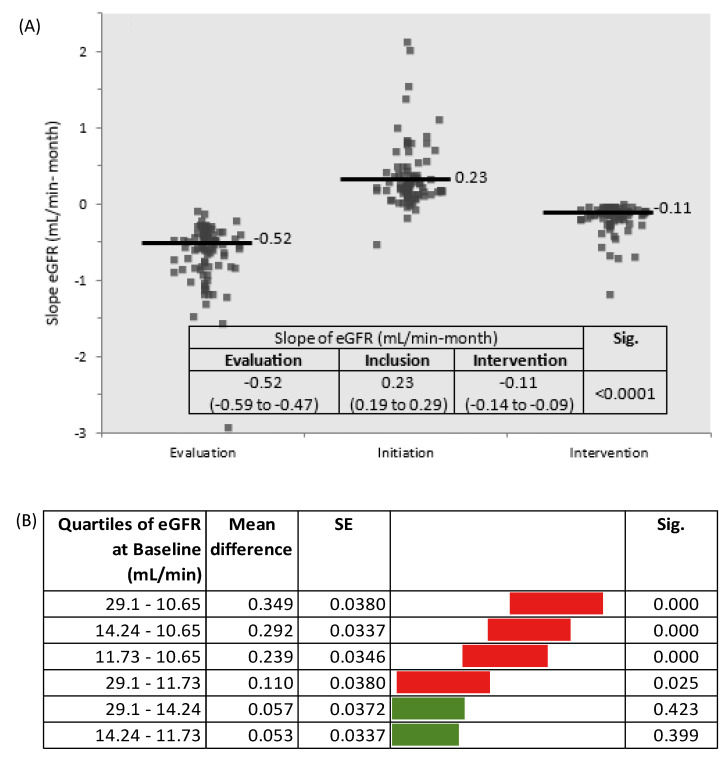
(**A**) Slopes of eGFR by study phases (Sig. between Evaluation and Inclusion phases and between Inclusion and Intervention phases); (**B**) Slopes of eGFR by quartiles of eGFR at baseline (Tukey–Kramer all pairs comparisons).

The slope of eGFR was −0.52 mL/min-month in the evaluation phase and about five-fold lower (−0.11 mL/min-month) during the intervention phase. Overall, during the intervention phase, eGFR decreased by only 1.5 mL/min per year (Figure 2 and Figure 3A; Table 1 and Table 2).

**Table 1:** Patients’ characteristics at Inclusion.

Demographic Characteristics	
Age (years)	61 (58 to 67)
Sex (male%)	66%
**Efficacy parameters**	
Proteinuria (g/g creatinine)	4.8(4.6 to 5.2)
eGFR (mL/min)	11.7 (11.2 to 12.2)
Mean arterial pressure (mmHg)	98 (93–102)
Mean arterial pressure <97 mmHg (%)	47
**Nitrogen balance**	
Urea (mg/dL)	127 (116 to 134)
Uric acid (mg/dL)	6.3 (6.2 to 6.4)
**Safety parameters**	
Body mass index (kg/m^2^)	27.3 (26.6 to 28.4)
Subjective global assessment A (%)	100
Serum albumin (g/dL)	3.9 (3.9 to 4.0)
C-reactive protein (mg/L)	13 (12 to 14)
Glycated hemoglobin (%)	8.5 (8.4 to 8.7)
**Adherence the diet**	
Estimated protein intake (g/kg/day)	0.89 (0.85 to 0.95)
Adherence to protein restriction (%)	5%
**Therapy**	
RAASi (%)	85%
Furosemide (%)	52%

Data are presented as median and 95% confidence interval (95% CI). eGFR—estimated glomerular filtration rate; RAASI—renin–angiotensin–aldosterone system inhibitors.

**Table 2:** The variations of investigated parameters in study phases.

	Baseline (*n* = 92)	End of Study (*n* = 92)	End of Study—Baseline Difference	Sig.
**Efficacy Parameters**				
Proteinuria (g/g creatinine)	5.2 (5.0 to 5.2)	1.6 (1.5 to 1.7)	−3.5 (−3.7 to −3.7)	<0.0001
Slope of proteinuria (g/g per mo.)	−0.3 (−0.32 to −0.28)			
eGFR (mL/min)	12.6 (11.7 to 13.1)	11 (10.3 to 11.5)	−1.5 (−1.7 to −1.2)	<0.0001
Slope of eGFR (mL/min per month)	−0.11 (−0.14 to −0.1)			
Mean arterial pressure (mmHg)	99 (90–109)	88 (85–88)	−11 (−17 to −7)	0.0002
Mean arterial pressure < 97 mmHg	47%	84%	6.0 (3.1 to 5.3) *	0.000
**Safety parameters**				
Body mass index (kg/m^2^)	27.1 (26.3 to 28.0)	26.0 (25.1 to 26.8)	−1.2 (−1.6 to −0.7)	0.004
Subjective global assessment A (%)	100%	100%	1(1 to 1) *	1
Serum albumin (g/dL)	3.9 (3.9 to 4.0)	4.1 (4.1 to 4.2)	0.2 (0.1 to 0.3)	<0.0001
C-reactive protein (mg/L)	14 (13 to 14)	9 (8 to 9)	−4.0 (−6.0 to −4.0)	<0.0001
Glycated hemoglobin (%)	8.1 (8.0 to 8.3)	8.1 (7.9 to 8.3)	−0.2 (−056 to −0.01)	0.04
**Nitrogen balance**				
Urea (mg/dL)	127 (116 to 134)	145 (133 to 149)	12 (12 to 15)	<0.0001
Uric acid (mg/dL)	4.4 (4.2 to 4.4)	4.4 (4.0 to 5.1)	−0.2 (−0.5 to 0.3)	0.47
**Mineral-bone disease parameters**				
Phosphate (mg/dL)	7.6 (7.3 to 8.1)	4.1 (3.6 to 4.6)	−4.1 (−4.6 to −3.6)	<0.0001
iPTH (pg/mL)	548 (537 to 553)	182 (174 to 195)	−370 (−370 to −370)	<0.0001
**Adherence the diet**				
Estimated protein intake (g/kg/day)	0.68 (0.67 to 0.69)	0.64 (0.63 to 0.63)	−0.03 (−0.05 to—0.01)	<0.0001
Slope of estimated protein intake (g/kg/day per month)	−0.03 (−0.05 to −0.01)			
Adherence to protein restriction (%)	39%	64%	2.9 (1.6 to 3.6) *	<0.0001
Estimated energy intake (kcal/kg/day)	31.3 (30.3 to 32.3)	30.5 (29.5 to 31.8)	−0.3 (−1.7 to 0.7)	0.23
Adherence to energy intake (%)	63%	65%	1.1 (0.6 to 1.9) *	0.8
**Therapy**				
RAASi (% patients)	100%	75%	0.01 (0.00 to 0.25) *	0.003
Furosemide (% patients)	62%	87%	4.1 (2.0 to 8.3) *	0.000

* Odd ratio. Data are presented as median and 95% confidence interval (95% CI). eGFR—estimated glomerular filtration rate; RAASi—Renin–angiotensin–aldosterone inhibitors.

The eGFR at initiation was indirectly related to the reduction in the rate of decline in kidney function (R^2^ 0.45; Beta −0.68; *p* = 0.000). Considering the eGFR slopes by quartiles of eGFR at baseline, the decline was higher in patients with eGFR < 14.2 mL/min (quartile 1–3) (Figure 3B).

#### 3.1.2. Proteinuria

During the intervention phase, proteinuria decreased about three-fold, by 3.5 g/g. The decrease was continuous (slope −0.3 g/g per month) without a cut-off level and was directly related to baseline proteinuria (R^2^ 0.70; *p* = 0.000) (Figure 2; Table 1 and Table 2).

**Figure 4:**
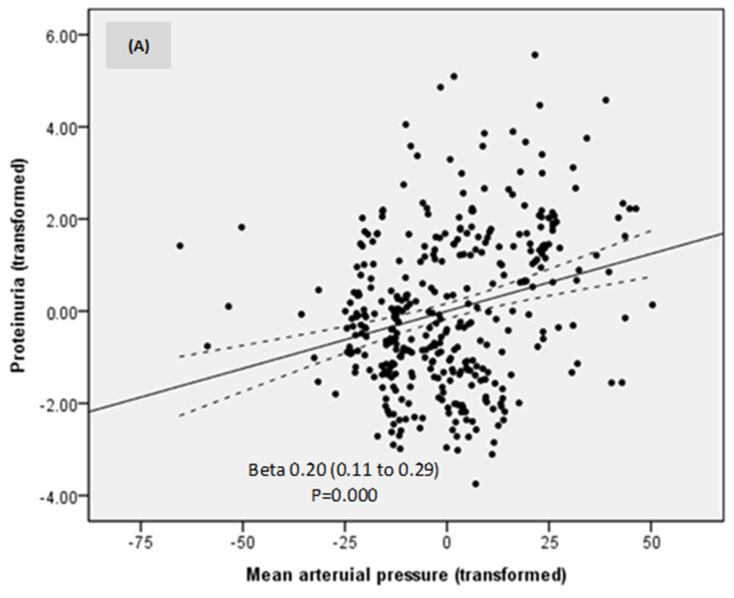

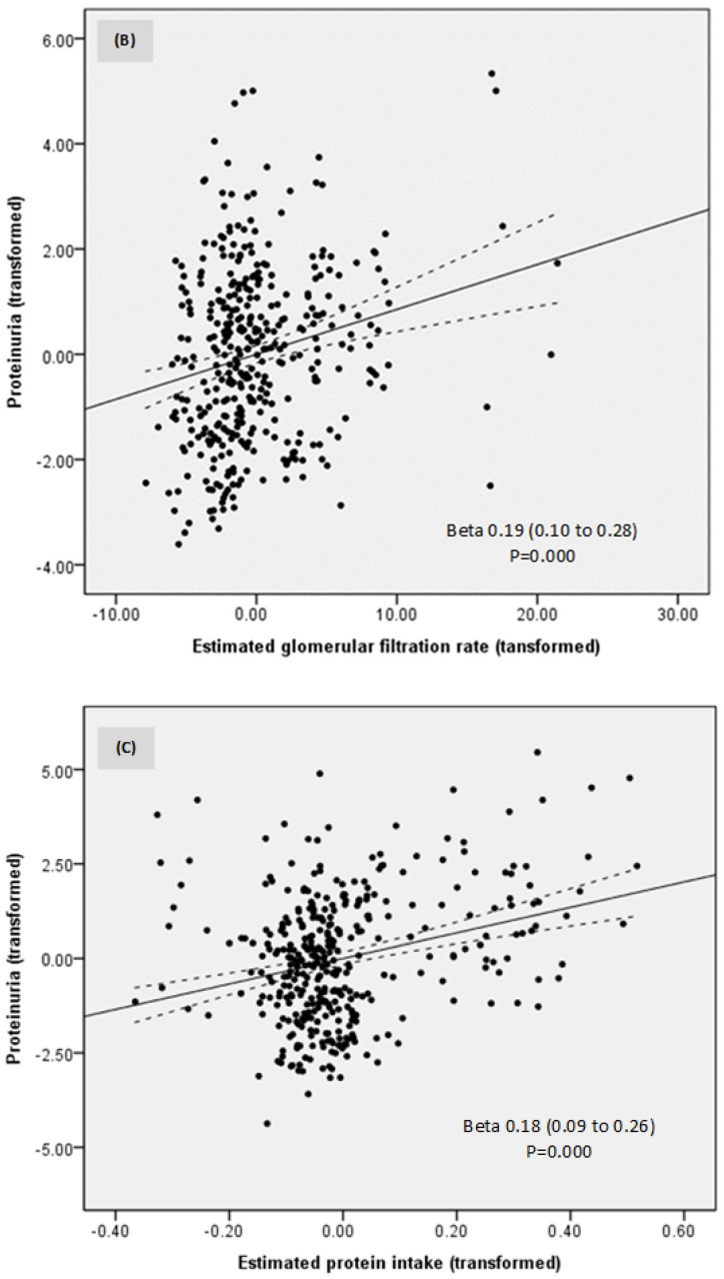
Relationships between proteinuria, mean arterial pressure (**A**), estimated glomerular filtration rate (**B**), and estimated protein intake (**C**).

In a model of linear regression, proteinuria was directly related to MAP, eGFR, and estimated protein intake (Table 3; Figure 4A–C). No other investigated parameter was related to proteinuria. Thus, lower proteinuria was predicted by lower MAP, eGFR, and estimated protein intake.

**Table 3:** Factors associated with proteinuria.

	B	SE	Beta	95% CI		Sig.
(Constant)	0.00	0.04		−0.09	0.09	1.000
Mean arterial pressure transformed	0.20	0.05	0.20	0.11	0.29	0.000
Estimated glomerular filtration rate	0.19	0.04	0.19	0.10	0.28	0.000
Estimated protein intake	0.18	0.05	0.18	0.09	0.26	0.000

Model of linear regression adjusted R^2^ = 0.22; *p* = 0.000; All variables were transformed to optimize accuracy. Dependent variable: Proteinuria.

#### 3.1.3. Nitrogen Balance

Uric acid levels remained stable during the study.

Although urea increased at EOS as compared to baseline, probably related to the decline in kidney function, no patient started KRT or died during the study period, supporting that nutritional intervention and meticulous monitoring and care of CKD complications are effective in safely postponing KRT even in high-risk DKD patients.

### 3.2. Safety Parameters

In the intervention phase, nutritional status and inflammation improved. Body mass index decreased by 1.2 kg/m^2^ but with no changes in the SGA score, while serum albumin increased by 0.2 g/dL and C-reactive protein decreased by 4 mg/L (Table 1 and Table 2).

Changes in serum albumin and CRP levels were closely related to proteinuria. Higher CRP levels were independently related to lower serum albumin levels (Beta −0.35; 95%CI −1.47 to −0.82) and to higher proteinuria (Beta 0.39; 95% CI 0.29 to 0.49) (adjusted R^2^ 0.34; *p* = 0.000).

Blood glucose control also improved during the intervention, as HbA1c slightly declined (0.11%) (Table 2).

### 3.3. Compliance to the Dietary Intervention

**Figure 5:**
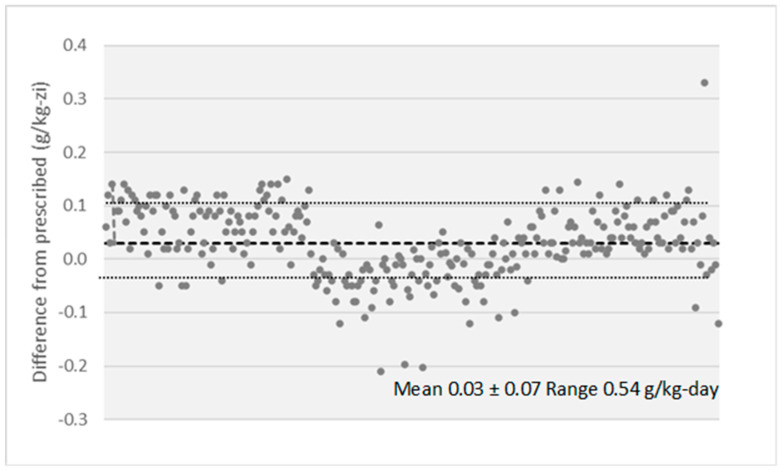
Difference between prescribed and estimated protein intake along the intervention phase.

The estimated protein intake decreased from inclusion (0.89 to g/kg/day) to baseline (0.68 g/kg/day) and to EOS (0.64 g/kg/day) (Table 2; Figure 2). In the intervention phase, the median difference between estimated protein intake and prescription was only 0.03 g protein/kg/day. A strict compliance with protein restriction was observed in 62% of measurements (Figure 5).

Although only 19 patients (21%) were strictly adherent (±10% of recommended) to the prescribed protein intake, the difference in estimated protein intake between adherent and not adherent patients was 0.02 g/kg/day. Moreover, when the decrease in proteinuria and slope of eGFR during the intervention phase were compared by adherence, no difference was observed (Table 4).

**Table 4:** Slopes of proteinuria and eGFR by adherence to protein intake.

Slope Bs—EOS	Adherence	Median	95% CI		Difference 95% CI			Sig.
Proteinuria (g/g-mo)	No	−0.29	−0.32	to −0.27	−0.02	−0.06	to 0.02	0.30
	Yes	−0.31	−0.37	to −0.27				
eGFR (mL/min-mo)	No	−0.11	−0.14	−0.09	0.01	−0.04	to 0.06	0.55
	Yes	−0.11	−0.21	−0.04				

Adherence to protein intake was defined as a more than ±10% variation from Recommended. Difference in estimated protein intake Adherent—Non-Adherent −0.02 (−0.03 to −0.01) g/kg/day *p* = 0.0030.

The estimated energy intake was close to the prescription (30 kcal/kg/day) and 68% of participants were adherent to the prescription at all study moments (Table 1 and Table 2).

## 4. Discussions

This study is one of the few investigating the effects of a low protein diet supplemented with ketoanalogues of essential amino acids on top of conventional nephroprotective therapy in high-risk type 2 diabetic patients with advanced CKD and heavy proteinuria. The main findings are an impressive three-fold reduction in proteinuria (3.5 g/g) and a five-fold reduction of eGFR slope to only 1.5 mL/min-year, close to that observed in the general population (1 mL/min per year). These improvements were directly related to a lower protein intake. Patients with eGFR < 14.2 mL/min at baseline had a higher decline in eGFR. However, none of them needed KRT initiation. Therefore, sLPD should be started when eGFR > 14 mL/min to slow down CKD progression, while in patients with Egfr < 14 mL/min the sLPD seems useful to postpone KRT, ameliorating the metabolic abnormalities.

Aiming to improve kidney outcome, low protein and very low protein diets, supplemented or not with ketoanalogues of essential amino acids were tested in non-diabetic 4-5 CKD patients with different results [9,11,23,24]. A recent meta-analysis concluded that very low protein diets (0.3 to 0.4 g/kg-day supplemented with KA reduce the number of non-diabetic CKD 4 or 5 participants who progress to ESKD as compared to those on low or normal protein diets. The sVLPD reduced the risk of ESKD with 36% but had no influence on the decline in eGFR. Effects on proteinuria were not reported [9].

Although diet is a key component of diabetes management, the clinical experience with LPD in patients with type 2 diabetes mellitus and advanced CKD is limited, partially because of difficulties to provide enough energy while reducing both carbohydrates and protein intake, but also because of the low acceptance of such diets by patients [25,26]. However, two meta-analyses evaluated the existing studies and concluded that low/very low protein diets are effective and safe in patients with DKD and suggested further research for confirmation [13,15].

We investigated patients with type 2 diabetes mellitus and advanced CKD (eGFR 11.7 mL/min) with heavy, nephrotic range, proteinuria (4.8 g/g creatinine). Notably, no other of the reported studies enrolled patients with nephrotic range proteinuria and only in a few eGFR was so low. A run-in phase was necessary for intensive nutritional counseling, implementation of diet and verification of adherence. The intervention consisted in a diet providing 0.6 g protein (mostly of vegetal origin)/kg-day, supplemented with KA, on top of conventional nephroprotective and diabetes therapy. To improve adherence, five meals with protein of animal origin were allowed in a week. The nephroprotective and anti-diabetic therapy was continued and adjusted along the 12-month intervention phase.

### 4.1. Efficacy Parameters

#### 4.1.1. Estimated Glomerular Filtration Rate

In the run-in phase, eGFR increased by 1.5 mL/min, probably related to the lower intake of creatine from animal meat (one portion of pork meat contains 1.5 g of creatine). In the intervention phase, the decline in eGFR was almost steady until month 12, when a significant decline was interrupted by a small increase (0.3 mL/min) from study month 6 to month 9, when the proportion of patients treated with RASSi decreased and the proportion of those treated with furosemide increased, aiming to prevent further decreases in eGFR and to control BP.

The decline in eGFR slope was 5-fold lower in the intervention phase as compared to the evaluation phase. Overall, eGFR decreased by only 1.5 mL/min-year during the intervention phase, close to the accepted decline in the general population (1 mL/min/year) but about 3-fold lower than expected in this high-risk DKD patient (4 mL/min per year) [27,28]. The slope of eGFR was −0.11 mL/min per month, a lower rate than reported by Barsotti et al. (−0.22 mL/min/month) in DKD and similar to that reported by Chauveau et al. (−0.15 mL/min/month) in CKD patients with heavy proteinuria [16,29]. Moreover, none of our high-risk DKD patients needed KRT in a 12-month period. Thus, sLPD/sVLPD could efficiently slow down the decline in eGFR and postpone KRT initiation even in patients with advanced DKD and heavy proteinuria.

Notably, the eGFR slope depended on the baseline eGFR. Subjects with eGFR > 14.2 mL/min at baseline had the slowest decline in eGFR. Although patients with eGFR < 14 mL/min benefited less in terms of CKD progression, no patients needed KRT, which suggests that, in this group, sLPD delayed KRT initiation mainly by relieving metabolic abnormalities of advanced CKD. Thus, more benefit on CKD progression would be reached starting sLPD at eGFR > 14 mL/min, as also observed by other authors.

#### 4.1.2. Proteinuria

Proteinuria promotes the progression of CKD, especially in patients with DM [5,18] and is related to hyperfiltration and podocytes lesions [5,18]. Hyperfiltration results initially from over-stimulation of sodium-glucose co-transport and thereafter form over-activation of SRAA. A higher animal protein intake also increases hyperfiltration. In time, hyperfiltration and glomerular hyperfiltration produce structural damage, epithelial–mesenchymal transition, and interstitial fibrosis, which accelerates CKD progression [30]. By reducing hyperfiltration, a low protein intake could attenuate all these processes.

In our patients, proteinuria impressively declined 3-fold, from 5.2 to 1.6 g/g, with 3.5 g/g. The decrease was directly related to baseline proteinuria. In other studies that evaluated the effect of sLPD in DKD, the decrease in proteinuria varied from 2.4 to 4.2 g/g and was also directly related to baseline proteinuria: the higher proteinuria at baseline, the higher its reduction [30,31]. In our experience, the magnitude of proteinuria reduction was higher, probably because baseline proteinuria was also higher than in the previous reports. Thus, DKD patients with higher proteinuria would benefit the most from sLPD.

When data were pooled in meta-analyses, LPD/VLPD, supplemented or not with KA, the median decrease in proteinuria was 1 g/g in one meta-analysis [32] but had no significant effect in another [13]. However, the results of the studies are difficult to compare, since the main determinants of proteinuria reduction, i.e., proteinuria and eGFR at baseline, as well as the duration of the nutritional intervention, largely differed.

Our data show a continuous decrease in proteinuria. Therefore, we could not find a cut-off level at any of the study moments, as found by Chauveau et al. [32]. A cut-off level would have been useful as a predictor of the response to the nutritional intervention.

When values at all study moments were analyzed, proteinuria was independently related to MAP, eGFR, and estimated protein intake.

A lower MAP was associated with lower proteinuria as expected, even if MAP variations were small in our patients, underlying the importance of blood pressure control in preventing DKD progression and supporting the actual guidelines and recommendations [3]. The beneficial effects of the dietary intervention on blood pressure control were discussed in our previous paper [17].

Proteinuria was also directly dependent on eGFR and protein intake, suggesting that the decrease in proteinuria was also driven by a reduction in both eGFR and protein intake. However, the rate of eGFR decline was limited to only 1.5 mL/min/year.

These could be explained by a decrease in hyperfiltration. As an important part of the kidney workload is allocated to the excretion of nitrogen waste products, ingestion of animal-derived proteins increases renal plasma flow and decreases renal vascular resistance, resulting in hyperfiltration, which increases proteinuria and eGFR [33]. Accordingly, lowering animal protein intake and switching from animal to vegetal-derived proteins are expected to reduce hyperfiltration, resulting in both proteinuria and eGFR decreases. Thus, LPD could reduce hyperfiltration through a hemodynamical pathway similar to RAASi. As our patients were treated with RAASi during most of the intervention phase, diet seems to act synergically with pharmacologic therapy in slowing down DKD progression through a hemodynamic effect [4,7]. However, the effects of RAASi were difficult to differentiate from those of LPD.

### 4.2. Safety Parameters

In this study, the nutritional parameters improved. Body mass index decreased without any change in SGA score, serum albumin increased, and glycemia control was ameliorated. C-reactive protein decreased in relation to the increase in serum albumin due to proteinuria reduction, even if the patients did not have full-blown nephrotic syndrome.

The decrease in BMI was in line with other reports and underlined in a meta-analysis [15]. In the context of DKD, the BMI reduction seems beneficial and not a sign of protein-energy malnutrition since it was associated with better control of glucose metabolism, as noted also by Bellizzi et al. [34].

Fears that a low-protein diet would increase the risk of protein-energy wasting exist [35,36]. Moreover, a recent analysis of the nutritional adequacy of LPDs found that both vegetarian and animal LPDs provide insufficient amounts of certain essential amino acids, oligo-elements and vitamins. Vegetarian diets providing less than 0.6 g/kg/day did not meet the recommended nutritional daily allowance [36]. Accordingly, the combined plant—animal protein diet supplemented with ketoanalogues of essential amino acids used in this study seems like a better option for advanced DKD. Such diets were also efficient and safe for the elderly with advanced CKD. Additionally, this type of diet seems to increase patients’ nutritional satisfaction and improve adherence to their nutritional plans [37].

A better control of CKD-mineral and bone metabolism parameters was noted in other studies [38], but further studies are needed.

In a one-year time-frame, no patient died from this cohort of high-risk CKD patients with DM. Thus, a supplemented low-protein diet with mixed vegetal and animal-origin diet seems safe in the long term.

### 4.3. Compliance with the Dietary Intervention

The median estimated protein intake was 0.89 g/kg/day at inclusion, lower than the median protein intake in the Romanian general population [39] but close to the upper limit recommended by KDIGO, probably due to the spontaneous reduction in protein intake observed in advanced CKD [40,41]. Therefore, the beneficial effects observed in this study support a higher reduction in protein intake in advanced DKD, as recommended by KDOQ [I7].

Low adherence to restricted protein diets, especially in the long term, was frequently reported and resulted in questionable beneficial effects of a dietary intervention [41,42]. In our patients, compliance with the diet was good. In the intervention phase, the estimated median protein intake was 0.63 g/kg/day (the median difference from the prescribed value was only 0.03 g/kg/day), and adherence was observed at 62% of measurements at all study moments.

Although only 21% of patients were entirely adherent to protein intake along the intervention phase, the median difference between adherent and not adherent was only 0.02 g/kg/day, too low to influence the diet effect on reductions in proteinuria and eGFR slopes. Accordingly, the change in the diet pattern seems more important than a minor deviation from the prescribed protein intake.

Another challenge is the low acceptance of such nutritional interventions. Of the 452 patients screened in this study, only 20% were eligible, accepted, and adherent to the diet.

The patients’ diet provided energy as recommended, allowing them to preserve their nutritional status. The estimated energy intake was around 30 kcal/kg/day at all study moments in 68% of participants.

In our experience, a key factor in improving adherence to a diet is a regular assessment of protein and energy intake by a 3-day food diary and permanent counseling, namely constant feedback from the medical team to the patients and their families. The permanent contact and exchange of data are a clue element to allow “on the way” changes in the diet and considerably increase adherence.

### 4.4. Limitations

This study has several limitations. Firstly, it was a single-center study, adding also a “center effect” since the experience in nutritional management of advanced CKD could increase both compliance and accuracy of data.

Secondly, aiming to ascertain the efficacy of the nutritional intervention, we included very high-risk type 2 diabetic patients with unusually high proteinuria for the eGFR level. As a biopsy was not performed because of advanced CKD, the exclusion of other primary diseases was based on a careful evaluation, including the presence of diabetic retinopathy. Moreover, only Caucasian patients with a good nutritional status, well-controlled blood pressure, and optimal compliance with the diet were included. Although the generalization of results may be limited, the conclusions can support the utility of dietary intervention in the management of advanced DKD in carefully selected, motivated, and monitored patients.

Thirdly, there was no control group. However, the period of observation was long, and the sample size is among the largest reported.

## 5. Conclusions

A mixed vegetal and animal low protein diet (0.6 g/kg/day) supplemented with ketoanalogues on top of conventional nephroprotective therapy was effective in reducing proteinuria (independently related to protein intake) and the decline in eGFR in high-risk CKD patients with type 2 diabetes mellitus. The diet attenuated both eGFR decline and CKD complications; moreover, when started at an eGFR of >14 mL/min.

This nutritional intervention was nutritionally safe. Although the initial acceptance of the diet was low, the adherence was good in the long term. Regular close evaluation of the dietary intake, continuous feedback, and contact with patients, families, and the medical team, as well as nutritional counseling, could change the dietary pattern and improve adherence.

Further studies are needed to confirm these results, strongly supporting the efficacy and safety of a low-protein diet as an important tool in the management of advanced diabetic kidney disease.

## Data Availability

The original contributions presented in the study are included in the article, further inquiries can be directed to the corresponding author.
